# A validation study of the Hungarian version of the Prediction of Alcohol Withdrawal Severity Scale: the role of kindling mechanism

**DOI:** 10.1192/j.eurpsy.2025.930

**Published:** 2025-08-26

**Authors:** O. Bagi, B. K. Kádár, F. F. Farkas, J. Gajdics, I. K. Pribék, B. A. Lázár

**Affiliations:** 1 Addiction Research Group, Department of Psychiatry, University of Szeged, Szeged, Hungary

## Abstract

**Introduction:**

The critical importance of preventing, recognizing early, and effective treatment of complicated form of alcohol withdrawal syndrome (cAWS) lies in its high mortality rate. cAWS include alcohol-related seizures (ARS), and delirium tremens (DT). The Prediction of Alcohol Withdrawal Severity Scale (PAWSS) was developed to identify patients at risk of developing cAWS. Recently, history of ARS and/or DT (“kindling mechanism”-related predictors) have been suggested as the strongest risk factor for developing cAWS.

**Objectives:**

The present study aimed to validate the Hungarian version of the PAWSS as suitable for evaluating the risk of developing cAWS and determining the significance of past ARS and/or DT occurrences.

**Methods:**

A total of 70 inpatients were enrolled from at the Department of Psychiatry, University of Szeged, Hungary in 2023 with a principal diagnosis of AWS. PAWSS, Severity of Alcohol Dependence Questionnaire (SADQ) and Alcohol Use Disorders Identification Test (AUDIT) were used. Demographic variables (age, sex) and clinical outcomes (development of cAWS) were collected. Statistical analyses were performed using Receiver Operating Characteristic (ROC) analysis, binary logistic regression analyses and chi-square test.

**Results:**

The ROC analysis showed that ≥ 6 is the optimal cutoff point in our sample. The sensitivity (73.91%), specificity (82.98) and positive- (68.00%) and negative- (86.67%) predictive values were highest for the threshold value of 6. In the first binary logistic regression model our results indicate that the PAWSS score of 6 or more was identified as a significant predictive factor for the current cAWS (OR = 12.332; 95% CI = 3.468–43.85; p < 0.001). The results of the second binary logistic regression showed that the history of the cAWS (OR = 6.811; 95% CI = 2.084–22.25; p = 0.001) and the SADQ total score (OR = 1.048; 95% CI = 1.001–1.10; p = 0.043) were significant predictive factors for the current cAWS. The chi-square test results showed significant difference between the rate of the history of cAWS and the current cAWS (χ2 (1) = 13.0; *p* < 0.001) and 21.4% (n = 15) of the patients had both current and previous cAWS. The Phi-coefficient was 0.431, which indicates that the history of complicated AWS has a relatively strong effect on the current cAWS.

**Conclusions:**

Our results revealed that the Hungarian version of PAWSS is a valid tool for predicting cAWS with a different cutoff score compared to the original version. Furthermore, our findings suggest that the risk of developing cAWS is independent of the severity of alcohol use disorder. Our results also demonstrated that a history of cAWS is a significant predictor in the development of future episodes of cAWS.

**Disclosure of Interest:**

None Declared

